# Spotlight on early-career researchers: an interview with Gregory Lavieu

**DOI:** 10.1038/s42003-018-0144-1

**Published:** 2018-09-26

## Abstract

This interview in our series highlighting early-career researchers is with Gregory Lavieu, INSERM investigator at Institut Curie in Paris, France. In this series, we aim to bring attention to the diversity and individual stories of early-career researchers (defined as postdoctoral scientists through to tenure, or the equivalent). Gregory joined the institute as a permanent researcher in October 2017 to unlock the mysteries of extracellular vesicles. Here he discusses the many unanswered questions in the field as well as his unconventional path to his current position.

**Figure:**
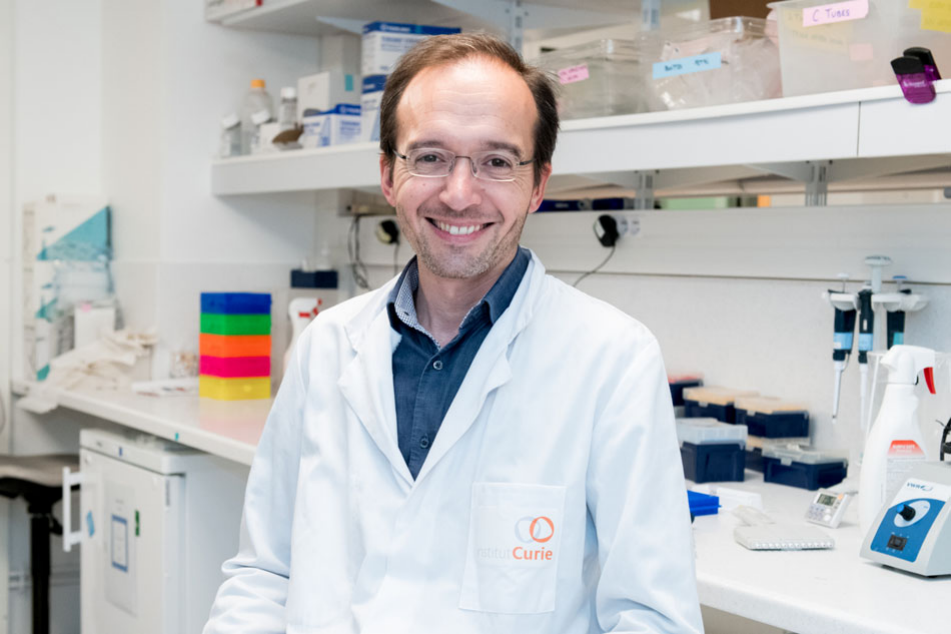
Image credit: Gregory Lavieu

Please tell us about your research interests.

After many years working on protein dynamics within the Golgi apparatus, I have decided to dedicate my research efforts toward understanding extracellular vesicles, especially the delivery process within acceptor cells. Over the last decade, extracellular vesicles (EVs) have been associated with multiple physiological functions, and most of them suggest the transfer of cytosolic molecules from donor to acceptor cells.

We still don’t understand the delivery mechanism. How do vesicles enter cells? Is it receptor-dependent? How do vesicles deliver their contents within the cytosol of the acceptor cells? Does it require membrane fusion? If yes, what is the nature of the target membrane and the fusion machinery? Those basic questions are not yet answered. This is not satisfying, especially considering how much we know about the cellular and molecular mechanisms that regulate the delivery of viruses or the transport of intracellular vesicles, which both share several physico-chemical properties with EVs.

In my research, I am capitalizing on my expertise on the biology and biochemistry of membrane trafficking. We are now developing cell-based and cell-free assays to test our hypotheses and later will use these to identify the machinery required for EV uptake and EV content delivery. I am particularly interested in the docking and fusion steps, that are likely to be required. This is very challenging, but also very exciting. From a mechanistic point of view, the EV field is relatively untouched and seems ripe for discoveries that will impact basic biology, and perhaps translational science, through the development of EV-mimetics as vectors for therapeutics delivery. But first, we must understand the cell biology of those vesicles!

What has your journey been to this point?

I have a long-standing interest in membrane trafficking. As a PhD student at Pierre and Marie Curie University, Paris, France, I first started to investigate the regulation of autophagy by sphingolipids, in the laboratory of Patrice Codogno. We were mostly investigating the cell signaling pathways that control autophagy and that were impacted by perturbations of the sphingolipid rheostat. Quickly, I became more attracted by the mechanical aspect of membrane trafficking. I was fortunate to encounter Stuart Moore, an excellent glyco-biochemist and experimentalist, who was leading a team within the research unit. Stuart was, and probably still is, an inspiration for young scientists with idealistic vision of the academic research career. He educated me about the reductionist approach and the outstanding progress that was made on the molecular aspect of the secretory pathway. While finishing my PhD on autophagy, I devoured the “classics” that revealed the core machinery of the secretory pathway and other membrane-related processes.

The next logical step was to contact Jim Rothman, a leader in the field. He offered me a post-doc position, and for the next 9 years or so, mostly at Yale University, we worked together on protein dynamics within the Golgi apparatus, a highly debated topic. I developed a synthetic approach to introduce, on purpose, perturbations of the Golgi within the cell, and test the different transport models that have been debated for years. We characterized a new transport mechanism that is dedicated to large cargoes that cannot fit into small transport vesicles budding from the Golgi cisternae. I termed this mechanism Rim Progression, which I believe reconciles several aspects of different transport models. However, this did not seal the debate about intra-Golgi transport, which keeps some of its mysteries.

At that moment EVs were gaining more and more attention, and I soon realized that this relatively unexplored field might be the ideal one to establish my independent research program. I had the technical expertise, the scientific culture permeated with outstanding discoveries made by our inspirational predecessors, and the desire to take a risky new scientific challenge. The only missing item was the location. Today, I am pleased to work as a permanent INSERM (French National Institute of Health and Medical Research) investigator at the Institut Curie, an outstanding place to do basic research. I am fortunate to work in the research unit led by Sebastian Amigorena, in close partnership with Clotilde Théry, who have both made significant contributions on EVs and immunity. This will surely contribute to catalyzing my research progress.

Can you speak of any challenges that you have overcome?

My career development was actually not as smooth and linear as the previous answer makes it sound. I had indeed difficulties to find an independent position. The outcome of my initial applications did not reach my expectations, and frankly, I was very disappointed.

So, against most of the advice I received, I decided to explore other opportunities and accepted a position in a California-based start-up company, which was developing a new microfluidic-based technology. There, I discovered a new environment, an outstanding technology, situational leadership, business development, and other sources of career motivations and satisfactions. Importantly, I was still committed to publishing our research, and I was glad to be one of the first to publish in your new journal.

This interlude was more than a pleasant way to turn my initial frustration into a very positive experience. Finally, the calls from basic research and my “beloved vesicles” were too strong and I decided to re-apply to academia, with a reinvigorated spirit. This time the outcome was much better and I joined the Institut Curie.

What are your predictions for your field in the near future?

The EV field needs two major breakthroughs. The first one is related to the physiology of EVs. What is still missing in the field is the identification of the principal physiological function (and perhaps an associated disease) for which EVs in general, or a particular subtype of EV, play an essential role. Among the known vectors of intercellular communication, secreted proteins such as hormones had insulin and glycemia regulation as a leading function, and lipoproteins had LDL and cholesterol regulation. Both vectors were associated with a clear and well characterized physiological function, which gave tremendous advantage to identifying the relevant cell or tissue models, and developing the appropriate assays. This ultimately has led to the discovery of the relevant molecular machineries. Today we know that EVs are involved in multiples functions, but there is no consensus regarding the main function of EVs. The identification of such a “champion” function for EVs would drive the field and channel research efforts toward the most relevant cell/tissue/organ models, and perhaps reveal the existence of a specific functional EV subtype.

This would greatly contribute to the second necessary breakthrough: the understanding of the mechanisms involved in EV-mediated transport, in particular EV-content delivery, for which I hope to significantly contribute in the near future.

What advice would you give to your younger self?

I do not like to either give or take advice. So, whatever I would suggest to myself, I would probably end-up doing the opposite.

Bonus question: What is the most amazing thing about exosomes that people may not know?

I like to quantify and do rough calculations to estimate the plausibility of a biological process. One example: the volume of cell cytoplasm has been estimated around 1 pL. Considering EVs as a 100 nm diameter sphere, equivalent to a volume of around 5 × 10^-7^ pL, it would take roughly 2 × 10^6^ EVs to fill/replace the cytoplasm of 1 cell. EV concentration in the blood is up to 10^9^ EVs per μL, a typical adult human has 5 L of blood, so there is approximately 5 × 10^15^ EVs in the blood per individual, which would in theory be enough to replenish the equivalent of 2.5 × 10^9^ cells. This represents 1/10,000 of the total blood circulating cells, and 50 times the number of blood circulating dendritic cells, a known target of EVs. This seems quite significant. Maths are here, we now need the Biology!

*This interview was conducted by Chief Editor Brooke LaFlamme*.

